# Protective Effect of *Aquilaria crassna* Leaf Extract against Benzo[a]pyrene-Induced Toxicity in Neuronal Cells and *Caenorhabditis elegans*: Possible Active Constituent Includes Clionasterol

**DOI:** 10.3390/nu15183985

**Published:** 2023-09-14

**Authors:** Nattaporn Pattarachotanant, Panthakarn Rangsinth, Watis Warayanon, George Pak-Heng Leung, Siriporn Chuchawankul, Anchalee Prasansuklab, Tewin Tencomnao

**Affiliations:** 1 Natural Products for Neuroprotection and Anti-Ageing (Neur-Age Natura) Research Unit, Chulalongkorn University, Bangkok 10330, Thailand; nat.ahs11@gmail.com (N.P.); watis.w@chula.ac.th (W.W.); 2 Department of Clinical Chemistry, Faculty of Allied Health Sciences, Chulalongkorn University, Bangkok 10330, Thailand; 3 Department of Pharmacology and Pharmacy, Li Ka Shing Faculty of Medicine, The University of Hong Kong, Hong Kong SAR, China; ptkrs@hku.hk (P.R.); gphleung@hku.hk (G.P.-H.L.); 4 Department of Transfusion Medicine and Clinical Microbiology, Faculty of Allied Health Sciences, Chulalongkorn University, Bangkok 10330, Thailand; siriporn.ch@chula.ac.th; 5 College of Public Health Sciences, Chulalongkorn University, Bangkok 10330, Thailand

**Keywords:** xenobiotic, aryl hydrocarbon receptor, CYP1A1, CYP35, hexokinase, cyclin D1, polycyclic aromatic hydrocarbon

## Abstract

*Aquilaria crassna* (AC) is a beneficial plant widely used to alleviate various health ailments. Nevertheless, the neuroprotection, antiaging, and xenobiotic detoxification against high benzo[a]pyrene induction have not been investigated. This study aimed to investigate the effects of ethanolic extract of AC leaves (ACEE) in vitro using SH-SY5Y cells and in vivo using *Caenorhabditis elegans* (*C. elegans*). Neuroprotective activities and cell cycle progression were studied using SH-SY5Y cells. Additionally, *C. elegans* was used to determine longevity, health span, and transcriptional analysis. Furthermore, ACEE possible active compounds were analyzed by gas chromatograph–mass spectrometry (GC-MS) analysis and the possible active compounds were evaluated using a molecular docking study. First, ACEE possessed neuroprotective effects by normalizing cell cycle progression via the regulation of AhR/CYP1A1/cyclin D1 pathway. Next, ACEE played a role in xenobiotic detoxification in high B[a]P-induced *C. elegans* by the amelioration of lifespan reduction, and body length and size decrease through the reduction in gene expression in hexokinase (hxk) and CYP35 pathway. Finally, phytochemicals of ACEE were identified and we uncovered that clionasterol was the possible active constituent in powerfully inhibiting both CYP1A1 and hexokinase II receptor. Essentially, ACEE was recognized as a potential alternative medicine to defend against high B[a]P effects on neurotoxicity and xenobiotic detoxification.

## 1. Introduction

*Aquilaria crassna* Pierre ex Lecomte (AC) is known by the name of agarwood or eagle wood. It is a highly precious fragrant plant and widely used as an ingredient in the traditional Chinese and Korean medicines. Likewise, it has different pharmacological activities, including the attenuation of cigarette smoke-induced inflammation and oxidative stress in airway epithelial cells [1], antiglycation, and antioxidant properties [2]. Our previous study has demonstrated that AC exerted neuroprotection against hyperglycemia-associated neurodegenerative diseases, healthspan, and longevity improvement in *Caenorhabditis elegans* (*C. elegans*) [3]. 

Neurodegenerative diseases (NDs) are characterized by neuron loss and the pathophysiological change in brain-associated disorders, including the loss of neuronal function and structure. Alzheimer’s disease and Parkinson’s disease are the most common NDs in the elderly population [4,5]. The interesting risk of NDs is an environmental pollutant: benzo[a]pyrene (B[a]P). B[a]P is one of the representatives of polycyclic aromatic hydrocarbons (PAHs). It is a product of industrial processes and is present in wood fires, vehicle exhaust, cigarette smoke, and food products, especially smoked and grilled foods. B[a]P is classified as a cancer-causing agent in humans [6,7,8,9]. The main sources of human B[a]P exposure are inhalation, contaminated food, and skin absorption [10,11]. The previous findings suggest that chronic B[a]P exposure could induce behavioral, neuropathological, and chemical change causing NDs [4,5]. The xenobiotic process of B[a]P is metabolized by cytochrome P450 (CYP) to a carcinogenic agent causing epigenotoxicity, neurotoxicity, and impairment of animals’ fertility [12,13,14]. For B[a]P’s metabolism in the current study, we investigated two prominent isomers of CYP, namely, CYP1A1 and CY35, in *C. elegans* lacking the classical CYP1A1 pathway [15,16]. 

Presently, B[a]P is regarded as a dangerous environmental pollutant that can cause serious health ailments, particularly brain tissue and neuronal damage. Based on previous studies, AC possesses multipotent pharmacological properties for human health. Nonetheless, the protective effect of AC on B[a]P-induced toxicity has not been clarified. Consequently, we have been studying the protective activity and underlying mechanisms of AC extract against B[a]P-induced neuronal cell damage and xenobiotic processing in human neuroblastoma SH-SY5Y cells and nematode *C. elegans*. Additionally, the investigation of phytochemicals in AC extract was performed, and the ability of those identified phytochemicals against CYP and hexokinase was also evaluated using an in silico approach. 

## 2. Materials and Methods

### 2.1. Plant Extraction

**Figure 1:**
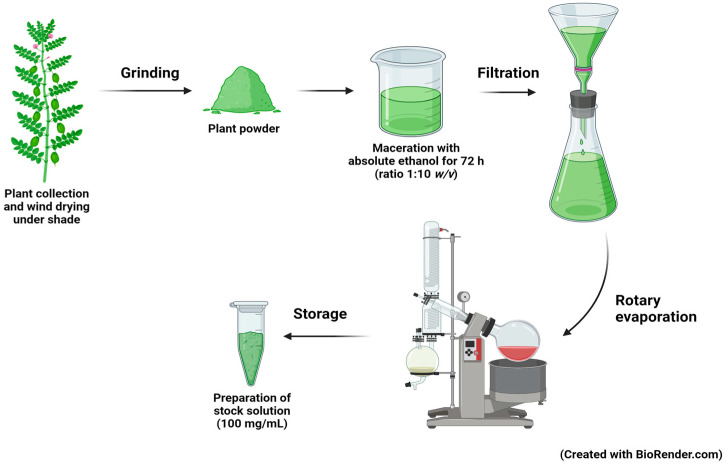
The extraction process of *Aquilaria crassna* (AC) leaves. Leaves of AC were collected, dried, grinded, and extracted with absolute ethanol using the maceration method. Ethanol was removed using rotary evaporation. The ethanolic extract of AC (ACEE) stock solution (100 mg/mL in DMSO) was prepared and kept at −20 °C.

Leaves of *Aquilaria crassna* Pierre ex Lecomte (AC) were collected from HRH Princess Maha Chakri Sirindhorn Herbal Garden, Rayong Province, Thailand. The plant was botanically identified and deposited at the herbarium of Kasin Suvatabhandhu, Department of Botany, Faculty of Science, Chulalongkorn University (Voucher specimen: A17634(BCU)). Plant material was extracted by the maceration method [17]. Briefly, the AC leaves were cleaned and dried under shade for several days at room temperature. After they had completely dried, the leaves were ground into a fine powder and then 40 g was extracted with 400 mL of absolute ethanol (ratio 1:10 *w*/*v*) at room temperature for 72 h. The resulting supernatant was collected, filtrated, and evaporated to dryness. Finally, the extract was prepared as a stock solution at the concentration of 100 mg/mL (as illustrated in Figure 1). The ethanolic extract of AC leaves was designated as ACEE. 

### 2.2. Gas Chromatograph–Mass Spectrometry (GC-MS) Analysis

Approximately 10 mg of ACEE extract was dissolved in 1 mL of absolute ethanol. The ethanolic extract was analyzed by GC-MS/MS to identify and estimate the relative abundance of organic substances. First, the chemical components of ACEE extract were separated by the GC (an Agilent 7890 series) technique. Then, GC coupled with an Agilent 7000C MS (Agilent Technologies, Inc., Santa Clara, CA, USA) and a capillary column (HP-5MS) worked very well for identifying unknowns. Finally, MS was used to measure the mass-to-charge ratio (*m*/*z*) of charged particles and determine the molecular weight, the chemical structures of molecules, and the elemental composition [17]. The obtained spectra were compared with NIST Mass Spectrometry Data Center to identify phytochemical constituents (https://www.sisweb.com/software/ms/wiley.htm) (accessed on 5 July 2023).

### 2.3. Antioxidant Determination

These assays including Folin-Ciocalteu phenol, total flavonoid, and radical scavenging activity were modified for a microplate format as previously described [18]. 

#### 2.3.1. Folin-Ciocalteu Phenol Assay

The total phenolic content was performed by the combination of (50 µL) ACEE or gallic acid (standard phenolic compound) solution and 10% Folin-Ciocalteau reagent (50 µL). This mixture was incubated in the dark at room temperature (RT) for 30 min. Having been incubated, sodium carbonate solution was added and the combination was left in the dark at (RT) for 20 min. The content of phenolic compounds was analyzed by measuring the reaction absorbance at 760 nm and expressed in a gallic acid equivalent (GE) mg/g of dry weight [19].

#### 2.3.2. Assay for Total Flavonoid Content

First, 50 µL of 1 mg/mL ACEE extract was mixed well with 150 µL of 95% ethanol. Then, the extract mixture was added with the solution of 10 µL of 1 M sodium acetate (NaOAc), and 10 µL of aluminum chloride (AlCl_3_). The combination was left in the dark at RT for 40 min and the reaction absorbance was measured at 415 nm. Total flavonoid content was expressed in a quercetin equivalent (QE) mg/g of dry weight [20,21].

#### 2.3.3. Radical Scavenging Activity Assays

Free radical scavenging activity was analyzed by using stable radical DPPH (DPPH•) [22] and stable cation radical ABTS (ABTS•+) [23]. The preparation of the working solution 2,2′-azinobis (3-ethylbenzthiazoline-6-sulphonic acid) (ABTS•+) (OD_734_ = 0.7–0.8) was carried out by the addition of 2.45 mM K_2_S_2_O_8_ to 7 mM ABTS (ratio 2:3) and the incubation of the working ABTS reagent at 4 °C for 16–18 h. Likewise, the working solution of 0.2 mM of 2,2-diphenyl-1-picryl-hydrazyl-hydrate (DPPH•) (OD_517_ = 0.8–0.9) was prepared by ethanol dilution. In the assays, the prepared working solution of DPPH• or ABTS•+ was added into the ACEE extract (1 mg/mL). Then, the mixture was incubated at RT for 15 or 30 min for DPPH• or ABTS•+, respectively. The absorbance of free radical scavenging activity was measured at 517 or 734 nm for DPPH• or ABTS•+. The antioxidant capacity was expressed in a vitamin C equivalent antioxidant capacity (VCEAC) in mg/g of dry weight. 

### 2.4. Cell Culture

A human neuroblastoma cell was used in this study including SH-SY5Y cells (cell line service, Heidelberg, Germany). Then, SHSY5Y cells were cultured in DMEM/high glucose (HyClone, Logan, UT, USA) supplemented with 10% fetal bovine serum and antibiotics including 100 U/mL penicillin and 100 µg/mL streptomycin (Thermo Scientific HyClone, Logan, UT, USA). Cells were maintained in the humidified incubator with 5% CO_2_ at 37 °C.

### 2.5. 3-(4,5-Dimethylthiazol-2-yl)-2,5-Diphenyltetrazolium Bromide Tetrazolium (MTT) Assay

Benzo[a]pyrene or B[a]P (C_20_H_12_, CAS number: 50-32-8, molecular weight: 252.31 g/mol) was purchased from Sigma-Aldrich (St. Louis, MO, USA). In this experiment, B[a]P of 0–40 µM and ACEE of 0–100 µg/mL were used to determine both the toxic concentration of B[a]P and nontoxic concentration of ACEE with SH-SY5Y cells. Then, to find out the protective dose of ACEE against B[a]P-induced cytotoxicity, the cells were cotreated with a toxic concentration (40 µM) of B[a]P and ACEE of 0–25 µg/mL for 48 h. Afterwards, 5 mg/mL of MTT reagent (BioBasic Inc., Markham, ON, Canada) was added to each well and incubated at 37 °C for 4 h to induce formazan product formation. Next, 10% SDS in 0.01 N HCl was added and the cells were incubated at 37 °C overnight. The amount of formazan is proportional to the number of alive cells [24]. 

### 2.6. Cell Cycle Analysis Assay

Cell cycle was analyzed by the assessment of DNA content distribution. Having been treated with either B[a]P or ACEE and incubated for 48 h, fixed cells were prepared by incubating with 70% ethanol at −20 °C for 2 h. Then, the cells were washed 3 times and stained with propidium iodide containing RNase A for 15 min in the dark. The percentages of cell population were analyzed using a BD FACSCalibur flow cytometer and BD CellQuest^TM^ Pro software version 4.0.2 (BD Biosciences, San Jose, CA, USA) [25].

### 2.7. Western Blot Analysis

Protein samples were mixed with a 2X Laemmli sample buffer (ratio 1:1) and heated at 95 °C for 10 min. Once heated, the protein samples were transferred to 10% sodium dodecyl sulfate-polyacrylamide gel electrophoresis (SDS-PAGE) and run for 1.5 h at 120 V. According to its size, the protein band had been separated. Protein molecules were blotted on polyvinylidene fluoride (PVDF) membranes and then blocked for an hour in the blocking solution containing 5% (*w*/*v*) nonfat milk and 0.05% Tween-20. Western blotting was permitted to stay overnight at 4 °C with primary antibodies including AH Receptor (1:5000, sc-133088, Santa Cruz Biotechnology, Dallas, TX, USA), cyclin D1 (1:5000, 92G2, cat#2978, Cell Signaling Technology, Beverly, MA, USA), CYP1A1 (1:5000, B-4, sc-393979, Santa Cruz Biotechnology), and β-actin (1:10,000, 13E5, cat#4970, Cell Signaling Technology). Either rabbit (cat#7074, Cell Signaling Technology) or mouse IgG (ab205719, Abcam PLC, Cambridge, UK) horseradish peroxidase-conjugated antibodies and chemiluminescence detection reagent were used to allow the visualization of bands. These protein bands were obtained using ImageJ software version 1.53 [25].

### 2.8. Cultivation of C. elegans

*C. elegans* (Bristol N2 strain) and *Escherichia coli* (OP50) were acquired from the Caenorhabditis Genetics Center (University of Minnesota, Twin Cities, MN, USA). *C. elegans* were cultivated on nematode growth medium (NGM) agar coated with *E. coli* OP50 with an OD_600_ of 1.0 and maintained at 20 °C [26]. 

### 2.9. C. elegans’ Lifespan Assay

L1 larvae were cultured on different NGM coated with 0.1% DMSO, 40 µM B[a]P, or cotreatment of 40 µM B[a]P with 5 µg/mL ACEE. At the L4 stage, new NGM was prepared and L4 worms were transferred to separate them from their progeny and to avoid starvation. The number of dead and live worms was counted and recorded every day until all worms were dead [26].

### 2.10. Measurement of C. elegans’ Body Size and Length

N2 synchronized L1 larvae were cultured on different NGM coated with B[a]P at 0, 100, 200, and 300 µM, and were allowed to grow into adult day 1. Later, a 10× objective lens of brightfield microscope was used to image adult day 1 worms in each group. Later, the software Motic Image Plus 3.0. was used to analyze the body length and size of the adult worms [26].

### 2.11. Quantitative Reverse Transcription PCR (RT-qPCR)

This assay was performed to understand the underlying signaling pathways that drive the biological effects of B[a]P and ACEE. For cell line, to identify the pathway by which ACEE ameliorates the effect of B[a]P on the induction of cell cycle arrest in neuronal cells, SH-SY5Y cells were treated with 40 µM B[a]P and 5 µg/mL ACEE and cultured for 48 h. As well, for *C. elegans*, L1 larvae stage worms were treated with 300 µM B[a]P and 5 µg/mL ACEE for 72 h into adult day 1 to investigate the pathway by which B[a]P stimulated toxicity and metabolism in *C. elegans.* Additionally, RNA from both SH-SY5Y and the worms was extracted by GENEzol^TM^ reagent (Geneaid Biotech Ltd., New Taipei City, Taiwan) following RNA extraction protocol procedure. Reverse transcription and the qPCR were accomplished in the recommended protocol of iTAQ universal SYBR green supermix (Bio-Rad Laboratories, Hercules, CA, USA) and CFX Real time PCR, respectively. The measured fluorescent signals revealed the PCR results. All primer sequences are shown in Table 1.

### 2.12. Molecular Docking

The X-ray crystallographic structures of cytochrome P450 1A1 (PDB ID: 4I8V) [30] and hexokinase 2 (PDB ID: 5HG1) [31] were obtained from the RCSB Protein Data Bank. The structures of the phytochemicals were retrieved from the PubChem database. Initially, proteins and ligands were prepared as described in our previous reports [32,33]. The docking analyses were performed using the Lamarckian Genetic Algorithm with default parameters by AutodockTools 1.5.6 (version 1.5.6) software (The Scripps Research Institute, San Diego, CA, USA). Later, the protein–ligand interaction studies were further visualized using the Discovery Studio Visualizer (BIOVIA, San Diego, CA, USA).

### 2.13. Statistical Analysis

All data were displayed as the mean ± standard error of the mean (SEM). Means were from at least 3 independent experiments. Prior to conducting the multiple comparison tests, the data were run to check for the normality and the homogeneity of variance. A one-way analysis of variance (ANOVA) followed by a post hoc Tukey test was performed for the data that met the assumptions of both the normal distribution and the homogeneity of variance (*p*-value ≥ 0.05). In case the assumptions had been violated, the data were then analyzed using a Kruskal–Wallis test followed by a post hoc Mann–Whitney test. The *p*-value < 0.05 was considered statistically significant.

## 3. Results

### 3.1. Phytochemical Constituents of ACEE

GC-MS/MS chromatogram of ACEE revealed 11 major peaks. Peaks of the phytochemical compounds in ACEE with five major constituents being squalene (23.1%), friedelan-3-one (12.76%), vitamin E (10.00%), neophytadiene (8.53%), lupenone (6.6%), and acetyleburicoic acid (6.05%). The components which corresponded to the peaks are shown in Table 2.

### 3.2. Antioxidant Properties and Total Phenolic and Flavonoid Contents

The ACEE extraction yield was 8.50% (*w*/*w*). Interestingly, ACEE contained 61.4 ± 0.90 mg GAE/g dry weight for total phenolic content and 20.61 ± 2.79 mg QE/g dry weight for total flavonoid content. ACEE had antioxidant capacities of 62.16 ± 2.39 mg of vitamin C equivalent antioxidant capacity (VCEAC)/g dry weight in DPPH and 81.97 ± 0.90 mg VCEAC/g dry weight in ABTS. Furthermore, ACEE at the concentration of 5 µg/mL possessed the free radical scavenging capacities of 49.09 ± 2.24% and 94.91 ± 0.95% against DPPH and ABTS radicals, respectively.

### 3.3. Effects of ACEE and B[a]P on Cell Viability

**Figure 2:**
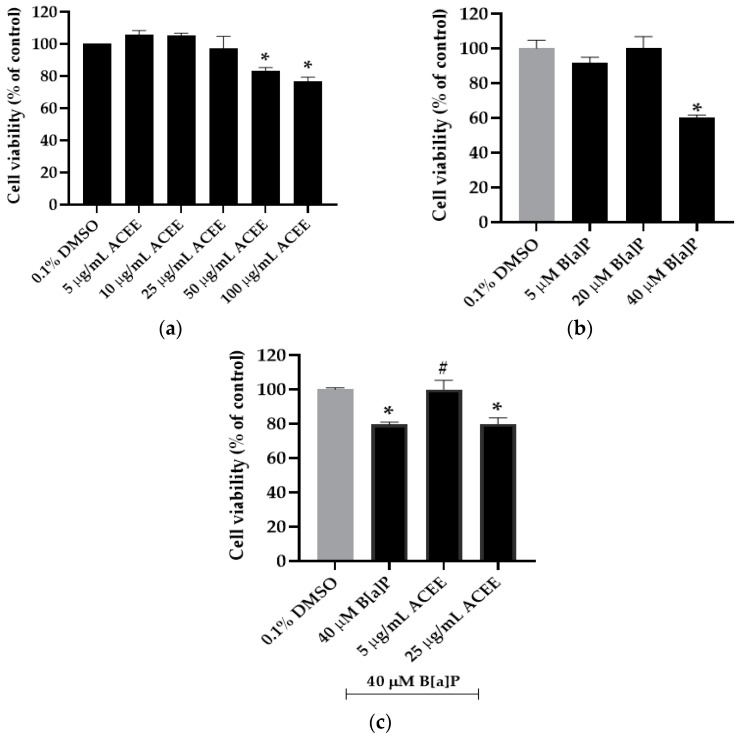
The effect of various concentrations of *Aquilaria crassna* ethanolic extract (ACEE) (**a**) and benzo[a]pyrene (B[a]P) (**b**) on SH-SY5Y cell viability was verified by 3-(4,5-Dimethylthiazol-2-yl)-2,5-Diphenyltetrazolium Bromide Tetrazolium (MTT) assay. In addition, MTT assay represented cell viability in cells pre-exposed with B[a]P and followed by ACEE treatment (**c**). Data are offered as the mean ± SEM, * *p* < 0.05 vs. control and ^#^ *p* < 0.05 vs. 40 µM B[a]P alone.

The nontoxic concentrations on cell viability of ACEE were at 5–25 µg/mL (Figure 2a). Cell viability was significantly decreased when cells were treated with ACEE at the concentration of 50 µg/mL (83.29 ± 1.98%, *p* value = 0.000) and 100 µg/mL (76.85 ± 2.45%, *p* value = 0.000) compared to the 0.1% DMSO control group. On the contrary, we found that B[a]P at the concentration of 40 µM could significantly decrease cell viability (60.08 ± 1.52% compared to the control group, *p* value = 0.000) (Figure 2b). Interestingly, only five µg/mL ACEE could notably exert the therapeutic effect of B[a]P-induced cell toxicity (Figure 2c). The effect of 5 µg/mL ACEE could improve cell viability in 40 µM B[a]P-treated cells from 79.56 ± 1.26% to 99.98 ± 5.36% (25.67% increase compared to B[a]P-exposed cells, *p* value = 0.007). Therefore, 40 µM B[a]P and 5 µg/mL ACEE were used for the further experiment.

### 3.4. Effect of ACEE on the Progression of Cell Cycle of SH-SY5Y Co-Cultured with B[a]P

**Figure 3:**
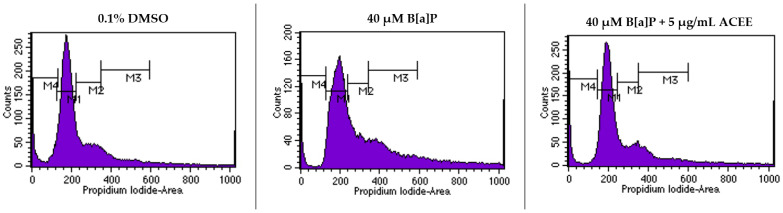
The effect of *Aquilaria crassna* ethanolic extract (ACEE) on the cell cycle progression. A flow cytometer was used to carry out the quantitative determination based on propidium iodide (PI) staining.

To further understand the protection of ACEE extract on B[a]P-induced cell cycle disturbance, a flow cytometer generated the data that showed the percentage of cells in the G0/G1 phase when treated with 40 µM B[a]P was significantly lower than in the 0.1% DMSO control group (a decrease of 18.08% when compared to the 0.1% DMSO control group, *p* value = 0.015). After treatment combined with 40 µM B[a]P and 5 µg/mL ACEE, the percentage of cells in the G0/G1 phase was significantly higher (an increase of 23.66% when compared to the group treated with 40 µM B[a]P alone, *p* value = 0.006). The flow cytometer results are shown in Figure 3 and Table 3. 

### 3.5. Effects of ACEE on B[a]P Disturbed Cell Cycle-Associated mRNA and Protein Expression

**Figure 4:**
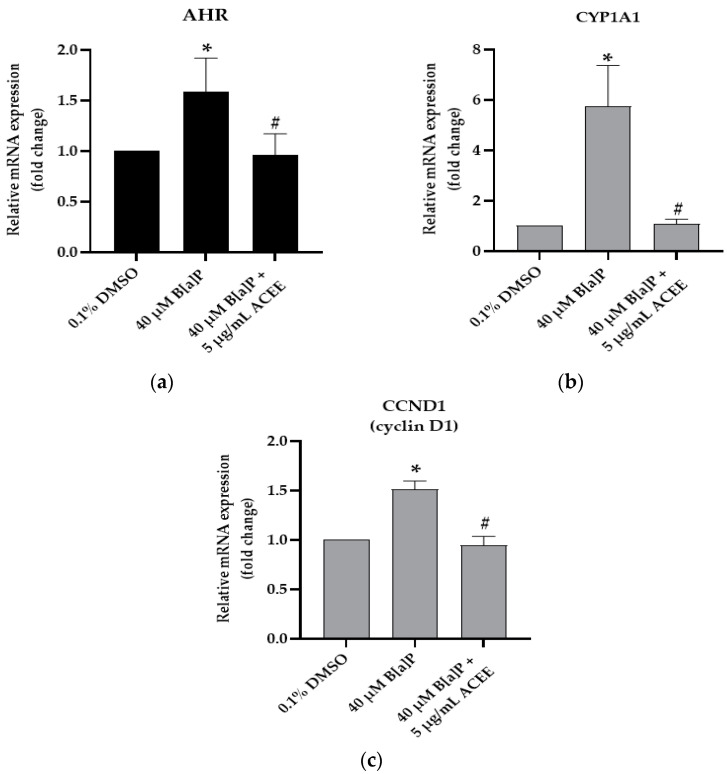
The effect of *Aquilaria crassna* ethanolic extract (ACEE) on cell cycle-associated gene expression in SH-SY5Y cells. Aryl hydrocarbon receptor (AHR) (**a**), cytochrome P450 1A1 (CYP1A1) (**b**), and cyclin D1 (CCND1) (**c**) relative mRNA expressions were quantified using Real Time qRT-PCR. All data are represented as the mean ± SEM, * *p* < 0.05 vs. control and ^#^ *p* < 0.05 vs. 40 µM B[a]P-treated group.

The results in Figure 4 indicate that 40 µM B[a]P disturbed cell cycle progression through significantly increasing the relative mRNA expression of AHR, CYP1A1, and cyclin D1 (1.62 ± 0.29-fold change (*p* value = 0.018), 5.75 ± 1.63-fold change (*p* value = 0.014), and 1.51 ± 0.08-fold change (*p* value = 0.019), respectively) in the B[a]P-treated SH-SY5Y cells compared with the 0.1% DMSO control group. After co-treatment with 40 µM B[a]P and 5 µg/mL ACEE, ACEE extract could attenuate the B[a]P effect and normalize the cell cycle progression. Hence, the relative mRNA expression of AHR, CYP1A1 and cyclin D1 in the ACEE-treated group was significantly decreased (^#^ *p* < 0.05 vs. 40 µM B[a]P alone), and the relative mRNA expression of AHR, CYP1A1 and cyclin D1 was 1.03 ± 0.17-fold change (*p* value = 0.023), 1.08 ± 0.19-fold change (*p* value = 0.034), and 0.94 ± 0.09-fold change (*p* value = 0.034) compared to the B[a]P-exposed group, respectively. 

**Figure 5:**
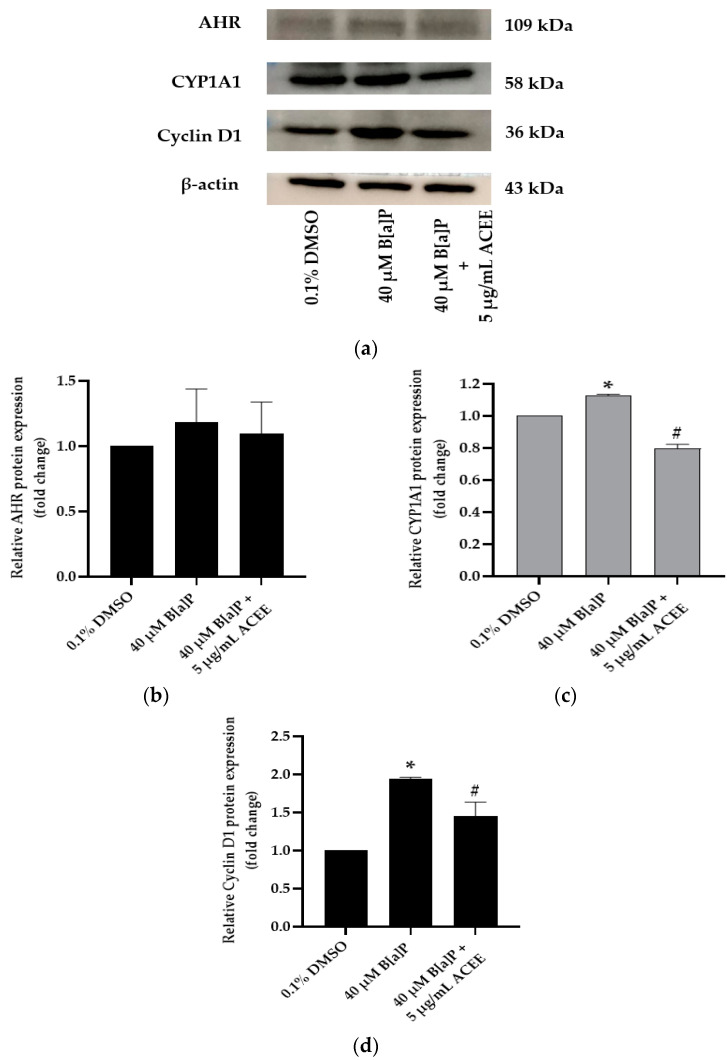
The effect of *Aquilaria crassna* ethanolic extract (ACEE) on cell cycle-associated protein expression in SH-SY5Y cells. Aryl hydrocarbon receptor (AHR), cytochrome P450 1A1 (CYP1A1), and cyclin D1 (cyclin D1) protein expressions are shown in representative Western blot images (**a**). Normalized values of AHR, CYP1A1, and cyclin D1 against β-actin (**b**–**d**), respectively. The mean ± SEM values of normalized AHR, CYP1A1, and cyclin D1 expression were obtained from three independent experiments, * *p* < 0.05 vs. control; ^#^ *p* < 0.05 vs. 40 µM B[a]P alone.

Moreover, Figure 5 shows the protein expression of CYP1A1 and cyclin D1 in SH-SY5Y cells was notably increased when cells were treated with 40 µM B[a]P alone (* *p* < 0.05 vs. control). The relative protein expression of CYP1A1 and cyclin D1 compared to the control group was 1.13 ± 0.01-fold change (*p* value = 0.019) and 1.94 ± 0.02-fold change (*p* value = 0.002), respectively. When treatment was combined with 40 µM B[a]P and 5 µg/mL ACEE, both CYP1A1 and cyclin D1 proteins were significantly decreased but only the AHR expression was not changed in response to the cotreatment with ACEE (^#^ *p* < 0.05 vs. 40 µM B[a]P alone). The relative protein expression of CYP1A1 and cyclin D1 compared to the 40 µM B[a]P-treated group was 0.79 ± 0.03-fold change (*p* value = 0.034) and 1.45 ± 0.18-fold change (*p* value = 0.043), respectively. 

### 3.6. Effects of ACEE on B[a]P-Reduced Lifespan of C. elegans

**Figure 6:**
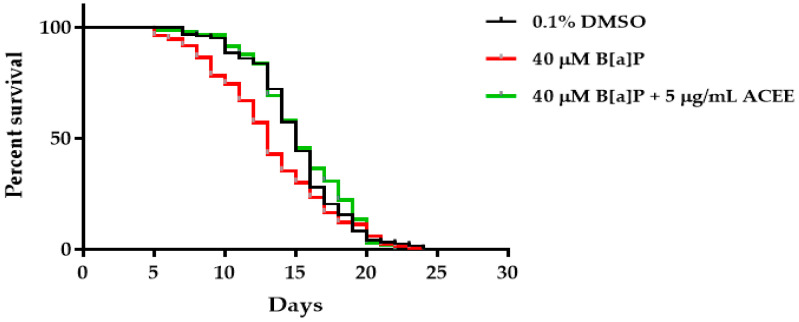
The effect of B[a]P and *Aquilaria crassna* ethanolic extract (ACEE) on *C. elegans* lifespan.

Our study indicates that high B[a]P-fed worms were manifested by shortened lifespan. The results in Table 4 reveal that the mean lifespan of 40 µM B[a]P-fed worms was shorter than that of the 0.1% DMSO control group (approximate 13.23%). Cotreatment with ACEE extract could also attenuate the B[a]P effect and increase the mean lifespan of high B[a]P-fed worms (13.38% in comparison to the mean lifespan of the B[a]P-treated group). All results are shown in Table 4 and Figure 6. 

### 3.7. Effect of ACEE on Toxicity and Metabolism of B[a]P in C. elegans

The toxicity of B[a]P was determined by body length assay. We found that all B[a]P at the concentrations of 100–400 µM could significantly reduce the body length compared to the 0.1% DMSO control group (0.77 ± 0.01-fold change, *p* = 0.000 for 100 B[a]P-treated group; 0.74 ± 0.01-fold change, *p* = 0.000 for 200 B[a]P-treated group; 0.77 ± 0.01-fold change, *p* = 0.000 for 300 B[a]P-treated group; 0.75 ± 0.01-fold change, *p* = 0.000 for 400 B[a]P-treated group). 

After the cotreatment of B[a]P and 5 µg/mL ACEE, ACEE could attenuate the toxic effect of B[a]P and cause the improvement in the body length (0.88 ± 0.01-fold change, *p* = 0.000 for 100 B[a]P-treated group; 0.83 ± 0.01-fold change, *p* = 0.000 for 200 B[a]P-treated group; 0.88 ± 0.01-fold change, *p* = 0.000 for 300 B[a]P-treated group). In contrast, ACEE activity on the body length improvement could not be detected in the 400 B[a]P-treated group.

**Figure 7:**
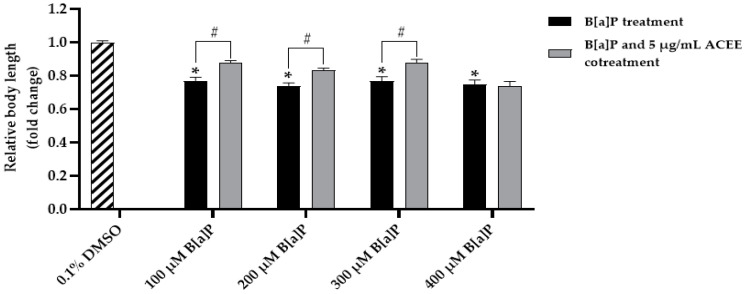
The effect of various doses of B[a]P and *Aquilaria crassna* ethanolic extract (ACEE) on body length. The mean ± SEM values of body length are shown, * *p* < 0.05 vs. control; ^#^ *p* < 0.05 vs. B[a]P-fed worms.

Therefore, the highest dose of B[a]P with which ACEE could exert the protective effect on B[a]P-induced body length reduction was 300 µM B[a]P (Figure 7). Based on this result, we determined to use 300 µM B[a]P as an inducer for toxicity and metabolism alteration of B[a]P in *C. elegans*.

**Figure 8:**
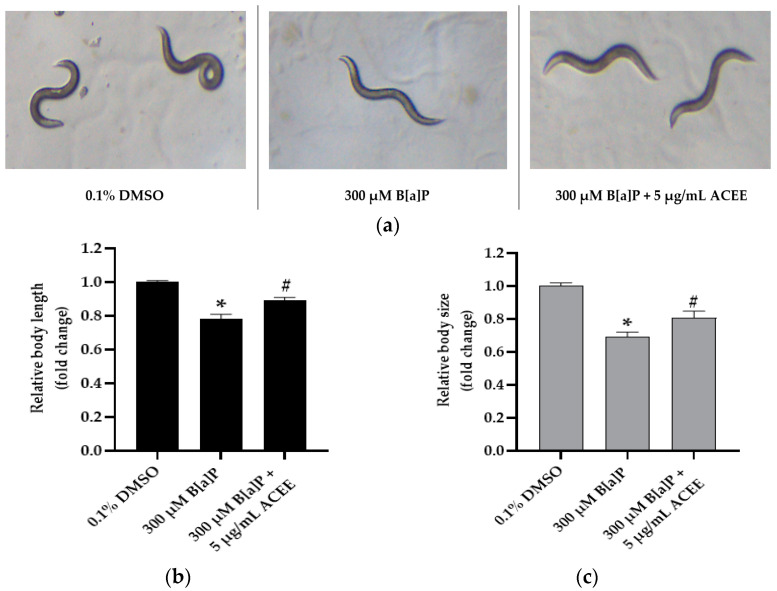
The effect of the ethanolic extract of *Aquilaria crassna* on B[a]P induced the reduction in body length and body size. Images were taken using a 10× objective lens of brightfield microscope, and representative images are shown (**a**) the mean ± SEM values of body length (**b**), and body size (**c**) are shown, * *p* < 0.05 vs. control; ^#^ *p* < 0.05 vs. 300 µM B[a]P-fed worms.

Not only body length assay, but as seen in Figure 8, we showed that 300 µM B[a] would significantly reduce both the body length and body size of worms in the B[a]P-treated group (0.78 ± 0.03-fold change (*p* value = 0.000) and 0.69 ± 0.03-fold change (*p* value = 0.000), respectively, * *p* < 0.05 vs. control). After cotreatment 300 B[a]P and 5 µg/mL ACEE, ACEE could attenuate the toxic effect of B[a]P and cause the improvement in the body length (0.89 ± 0.02-fold change (*p* value = 0.000); Figure 8b) and body size of adult day 1 worms (0.80 ± 0.04-fold change (*p* value = 0.000); Figure 8c) compared with B[a]P-treated group (^#^ *p* < 0.05 vs. 300 µM B[a]P alone).

### 3.8. Effect of ACEE on mRNA Expression of Cytochrome P450 35 Family (cyp-35) and Hexokinase (hxk) Genes in C. elegans

To identify the protective potential of ACEE on xenobiotic processing, mRNA expression of eight representative genes of the CYP35 family (*cyp-35A1, cyp-35A2, cyp-35A3, cyp-35A4, cyp-35A5, cyp-35B2, cyp-35B3 and cyp-35C1*) was studied.

**Figure 9:**
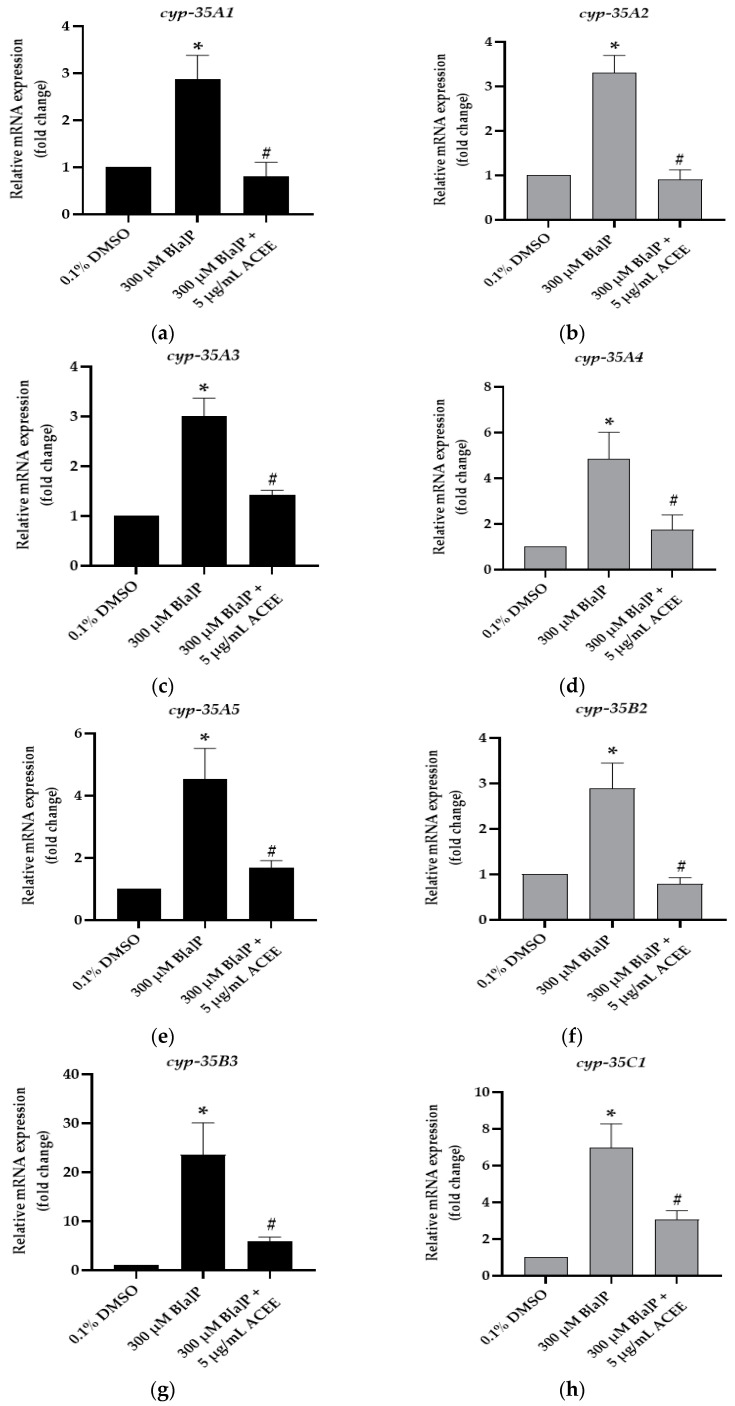
The effect of the ethanolic extract of *Aquilaria crassna* on B[a]P-induced cytochrome P450 35 family (*cyp-35*) mRNA expression. The relative mRNA expression of (**a**) *cyp-35A1*, (**b**) *cyp-35A2*, (**c**) *cyp-35A3*, (**d**) *cyp-35A4*, (**e**) *cyp-35A5*, (**f**) *cyp-35B2*, (**g**) *cyp-35B3*, and (**h**) *cyp-35C1* in B[a]P-fed worms following the treatments. The mean ± SEM values of mRNA expression are shown, * *p* < 0.05 vs. control; ^#^ *p* < 0.05 vs. 300 µM B[a]P-fed worms.

We found that 300 µM B[a]P could significantly increase the mRNA expression of all *cyp-35* genes in adult day worms. In the 300 µM B[a]P-fed group (Figure 9), mRNA expression was 2.87 ± 0.52-fold change, *p* value = 0.028 for *cyp-35A1*; 3.30 ± 0.40-fold change, *p* value = 0.014 for *cyp-35A2*; 3.01 ± 0.36-fold change, *p* value = 0.037 for *cyp-35A3*; 4.83 ± 1.18-fold change, *p* value = 0.014 for *cyp-35A4*; 4.53 ± 0.99-fold change, *p* value = 0.037 for *cyp*-35A5; 2.90 ± 0.55-fold change, *p* value = 0.014 for *cyp-35B2*; 23.59 ± 6.48-fold change, *p* value = 0.019 for *cyp-35B3*; 6.96 ± 1.32-fold change, *p* value = 0.005 for *cyp-35C1* (* *p* < 0.05 vs. control). As the treatment combined B[a]P with ACEE, the ACEE could significantly reduce mRNA expression compared with B[a]P-fed worms. mRNA expression was 0.80 ± 0.30-fold change, *p* value = 0.034 for *cyp-35A1*; 0.91 ± 0.21-fold change, *p* value = 0.021 for *cyp-35A2*; 1.42 ± 0.11-fold change, *p* value = 0.034 for *cyp-35A3*; 1.74 ± 0.65-fold change, *p* value = 0.043 for *cyp-35A4*; 1.68 ± 0.23-fold change, *p* value = 0.034 for *cyp-35A5*; 0.79 ± 0.13-fold change, *p* value = 0.021 for *cyp-35B2*; 5.94 ± 0.81-fold change, *p* value = 0.034 for *cyp-35B3;* 3.08 ± 0.46-fold change, *p* value = 0.034 for *cyp-35C1* (^#^ *p* < 0.05 vs. 300 µM B[a]P-fed worms). 

**Figure 10:**
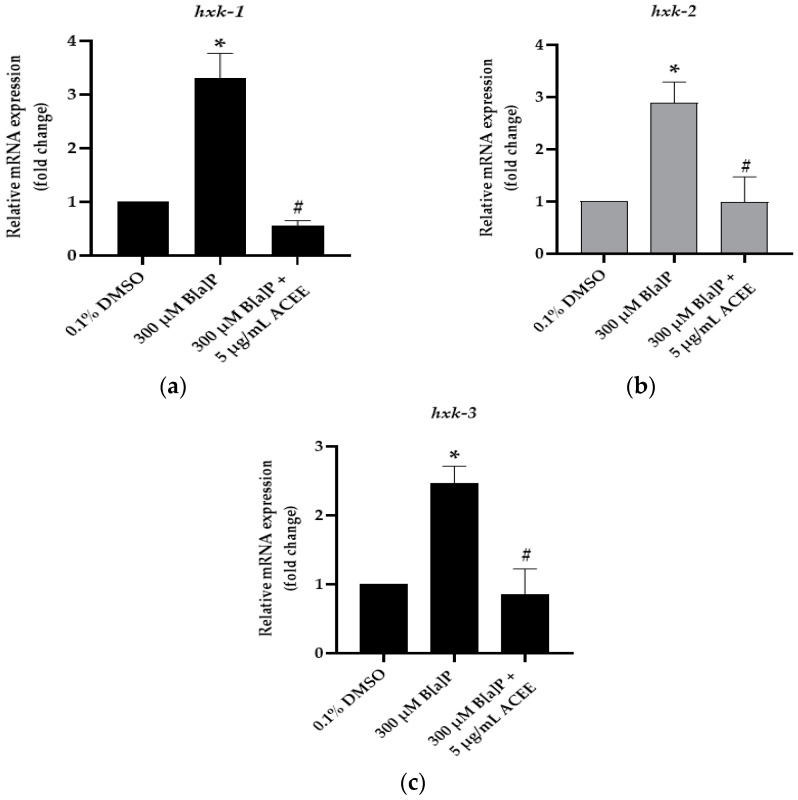
The effect of the ethanolic extract of *Aquilaria crassna* on B[a]P-induced hexokinase (*hxk*) mRNA expression. The relative mRNA expression of (**a**) *hxk-1*, (**b**) *hxk-2*, and (**c**) *hxk-3* in B[a]P-fed worms following the treatments. *The* mean ± SEM values of mRNA expression are shown, * *p* < 0.05 vs. control; ^#^ *p* < 0.05 vs. 300 µM B[a]P-fed worms.

In this study, we aimed to investigate the effect of B[a]P and ACEE on mRNA expression of *hxk* genes (Figure 10). We found that in the B[a]P-fed group, the significant increase in mRNA levels compared to the control group of *hxk-1*, *hxk-2* and *hxk-3* was 3.31 ± 0.46-fold change (*p* value = 0.014), 2.89 ± 0.4-fold change (*p* value = 0.024), and 2.46 ± 0.25-fold change (*p* value = 0.015), respectively (* *p* < 0.05 vs. control). mRNA levels of *hxk-1*, *hxk-2,* and *hxk-3* in the ACEE extract-treated group were lower than those in the B[a]P-treated group. The fold change of mRNA levels was 0.55 ± 0.1-fold change (*p* value = 0.034) for *hxk-1*, 0.98 ± 0.49-fold change (*p* value = 0.024) for *hxk-2,* and 0.86 ± 0.36-fold change (*p* value = 0.01) for *hxk-3* (^#^ *p* < 0.05 vs. 300 µM B[a]P-fed worms). 

### 3.9. The Ability of ACEE-Derived Phytochemical Constituents as Inhibitors of CYP1A1 and Hexokinase II Using an In Silico Approach

To further identify the competence of ACEE on CYP1A1 and hexokinase II, we used alizarin and purpurin as positive control for CYP1A1 [34], and metrizamide and lonidamine for hexokinase II [35,36,37]. As shown in Table 5, for the inhibition of CYP1A1, the binding energy was found to be −10.16, −8.56, and −8.97 kcal/mol for original ligand, alizarin, and purpurin, respectively.

**Figure 11:**
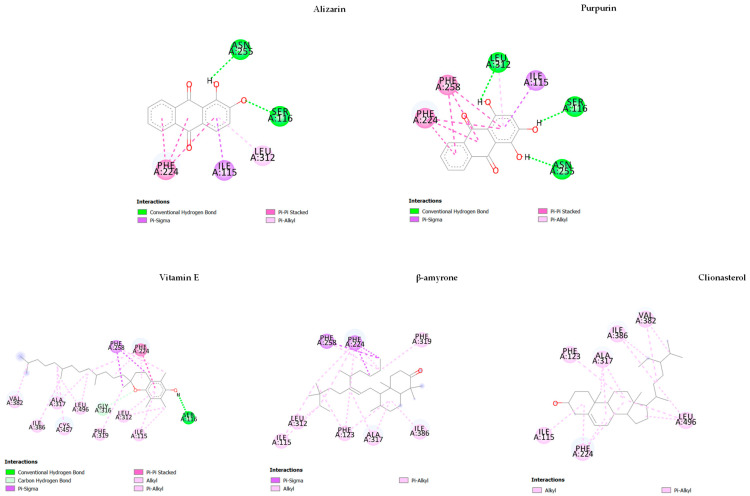
The results of the molecular docking study of cytochrome P450 1A1 (CYP1A1) (4I8V) are represented by the 2D diagrams of phytochemical–receptor interactions.

Based on the docking results in Table 5 and Figure 11, we found that only three phytochemicals, including vitamin E (−10.0 kcal/mol), β-amyrone (−10.12 kcal/mol), and clionasterol (−10.45 kcal/mol), exerted the outstanding inhibition against CYP1A1 with higher energy binding compared to the others and both positive controls, alizarin and purpurin. Prominently, clionasterol might be a strong inhibitor against CYP1A1 with the highest binding energy.

**Figure 12:**
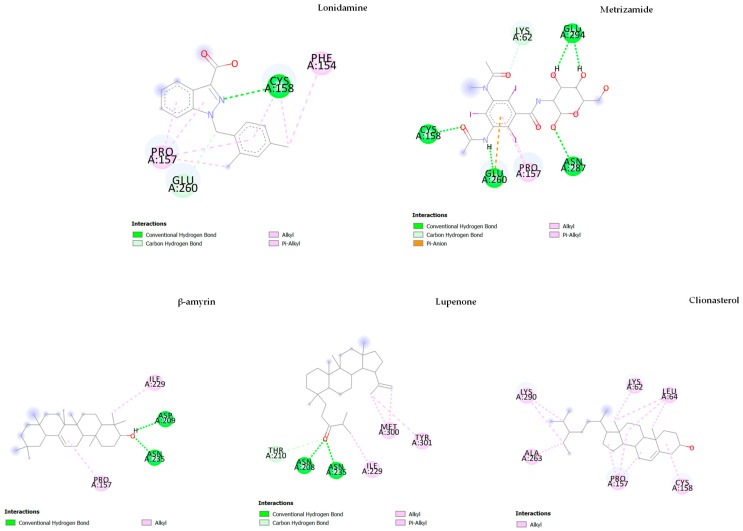
The results of the molecular docking study of hexokinase II (5HG1) are represented by the 2D diagrams of phytochemical–receptor interactions.

Additionally, as shown in Table 6 and Figure 12, for the inhibition of hexokinase II, the binding energy was found to be −6.52, −6.53, and −4.58 kcal/mol for original ligand, metrizamide, and lonidamine, respectively.

We found that the other three phytochemicals, including β-amyrin (−7.46 kcal/mol), lupenone (−7.05 kcal/mol), and clionasterol (−6.64 kcal/mol), showed strong inhibition against hexokinase II with the higher binding energy (higher than −6.53 kcal/mol of positive control metrizamide).

## 4. Discussion

Benzo[a]pyrene or B[a]P is a carcinogen and mostly discovered in cigarette smoke, vehicle exhausts, crude oil, coal tar, tobacco, and cooked food, especially grilled meats [38]. B[a]P may be one of the main causes of neurodegenerative diseases. The neurotoxicity effect of B[a]P on cognition, learning, and memory has been reported [39]. The neurotoxicity of B[a]P is linked to several metabolites through the activation of aryl hydrocarbon receptor and cytochrome P450 (CYP) system. Aryl hydrocarbon receptor (AHR) is a receptor frequently found in multiple organs such as the brain, liver, gastrointestinal tract, and lungs [40]. This receptor plays an important role in the control of xenobiotic detoxification through inducing CYP. An activated AHR translocates to the nucleus and dimerizes with an aryl hydrocarbon receptor nuclear translocator. Once dimerized, AHR binds to xenobiotic-responsive units to increase expression of CYP, mainly CYP1A1 [40]. Likewise, our current report confirmed that the ethanolic extract of *Aquilaria crassna* (ACEE) exerted the neuroprotective effect against the increase in cyclin D1 in B[a]P-induced neurotoxicity through an AHR-mediated CYP1A1 pathway. Interestingly, cyclin D1 is an influential protein of cell cycle activator that directs the progression of cell cycle and is upregulated in neurotrauma or in traumatic brain injury [41]. 

To further understand the effect of B[a]P and ACEE on organism survival, *C. elegans* were used as in vivo model. CYP35 family plays a crucial role in the xenobiotic processing in *C. elegans* lacking classical CYP1A1 enzyme [15]. In this study, the transcriptional analysis revealed that B[a]P could induce the expression of all eight *cyp-35* genes. Previous reports have linked *cyp-35A1*, *cyp-35A2*, *cyp-35A3*, and *cyp-35A5* to the toxic effect of B[a]P including lipid metabolism and alteration in longevity [42]. *C. elegans* worms lacking either *cyp-35A1* or *cyp-35A3* were characterized by an absence of a B[a]P-induced increase in toxicity and lipid metabolism [43,44,45]. Moreover, *C. elegans cyp-35A2*, *cyp-35A3*, or *cyp-35A5* mutants had no alteration of lifespan after B[a]P exposure [42]. In addition, *cyp-35A4*, *cyp-35B3*, and *cyp-35C1* genes play an important role in toxicity, change in lifespan, and metabolism in *C. elegans* after 3-bromopyruvate (3-BrPA) exposure [29]. Finally, *cyp-35B2* gene is involved in detoxification, the protection of dopaminergic neurodegeneration, and the maintenance of neuronal health in *C. elegans* after bacterial metabolite exposure [46]. These reasons are consistent with our current results. We found that B[a]P could cause changes in lifespan, and body length and size through the increase in *cyp-35* expression in B[a]P-fed worms.

Additionally, hexokinase (hxk) is an enzyme that plays a role in glucose homeostasis and organism development. Hxk is an enzyme catalyzing almost all hexasaccharides and producing glucose-6-phosphate (G6P) and energy through glycolysis [47,48]. In normal tissue, the activity of hxk is limited by low energy demand and the level of its metabolite G6P [49]. In a previous study, the high-level expressions of *hxk-1*, *hxk-2*, and *hxk-3* were identified in *C. elegans* exposed to 3-Bromopyruvate (3-BrPA). The high expression of these genes might be responding to the large ATP consumption during 3-BrPA metabolism [29]. Likewise, in this study, we found that high B[a]P could enhance the expression of *hxk-1*, *hxk-2*, and *hxk-3*. Conserved from *C. elegans* to mammals, the previous report indicated that *hxk-2* and *hxk-3* of *C. elegans* are closely related to hexokinase II in mammals [29].

Based on our DPPH and ABTS assays, ACEE exhibited free radical scavenging activity which may lead to the potential biological properties in antioxidant and anti-inflammation. In addition, correlating the effects of ACEE with the biological activities of its bioactive compounds revealed by the GC-MS are shown in Table 7.

Furthermore, the binding affinity of these phytochemicals of ACEE against CYP1A1 and hexokinase II was analyzed by docking analysis. The results presented that clionasterol might be the most potent inhibitor of both CYP1A1 and hexokinase II with the higher binding energy compared to positive control. In the previous study, clionasterol was one of drug-likeness ingredient found in traditional Chinese medicine called Bushen-Tiansui formula. This formula could improve the cognitive function of Alzheimer’s disease through CYP1A1 and CYP3A4 metabolism-related targets [80].

Overall, the findings from this present study support that ACEE could provide a neuroprotective effect against high B[a]P-induced neurotoxicity in human neuronal SH-SY5Y cells by the induction of cell cycle progression. Likewise, ACEE could attenuate the effect of B[a]P on shortening *C. elegans* lifespan, and body length and size through reducing the mRNA expression of AHR-responsive genes *cyp-35* and *hxk* genes. The current study demonstrates the effects of B[a]P and ACEE in both neuronal cell line and animal models. Further analysis of the dose-response relationship could be performed using the linear-quadratic dose-response model in order to better understand and predict the cellular response to B[a]P or ACEE exposure. Collectively, ACEE might be an effective neuroprotectant and detoxification agent against B[a]P exposure with a possible active constituent including clionasterol.

## 5. Conclusions

In summary, the AC leaf is composed of rich bioactive compounds and antioxidant properties. ACEE could represent neuroprotectant from B[a]P-induced neurotoxicity. ACEE can protect neuronal cells from B[a]P-induced cell damage, including the induction of cell cycle normalization through the AHR/CYP1A1/CCND1 signaling pathway. ACEE also utilizes lifespan extension and xenobiotic detoxification via the *cyp-35* and *hxk* pathway. Additionally, clionasterol might be the prominent phytochemical that inhibits CYP-induced B[a]P’s metabolism into toxic metabolites. Essentially, ACEE could be advanced as an agent for the protection of B[a]P-induced xenobiotic toxicity in neuronal cells and *C. elegans*.

## Figures and Tables

**Table 1:** Primer sequences for the quantitative polymerase chain reaction (qPCR).

Primer Sequences for SH-SY5Y			
Gene	Forward Primer (5′→3′)	Reverse Primer (5′→3′)	Ref.
ACTB	GGCATCCTCACCCTGAAGTA	AGCCTGGATAGCAACGTACA	*
CCND1	ATGTTCGTGGCCTCTAAGATGA	CAGGTTCCACTTGAGCTTGTTC	[27]
AHR	GTCGTCTAAGGTGTCTGCTGGA	CGCAAACAAAGCCAACTGAGGTG	[28]
CYP1A1	GATTGAGCACTGTCAGGAGAAGC	ATGAGGCTCCAGGAGATAGCAG	
**Primer Sequences for *C. elegans***			
Gene	Forward primer (5′→3′)	Reverse primer (5′→3′)	Ref.
*act-1*	AGACAATGGATCCGGAATGT	CATCCCAGTTGGTGACGATA	[3]
*cyp-35A2*	GCATCCATTCTTAACGTCAGCTTC	ATCTTCGGTAACCTTCTCCTTCG	[29]
*cyp-35A4*	ACCAAATCAAGTCTGGGAGGTA	TCTTACTGACCGTGCTTCAACTC	
*cyp-35A5*	CATCTTCACCTTGTGGGTTGG	TTAGAAATATGGGCTTCGGGAAGG	
*cyp-35B3*	GTGATTATGAAACGTCGCAAGAAG	GCGGATGCTGTAAATGGAAAGAC	
*hxk-1*	GGAGAGTGTGCCCGAGTTGT	ATGCTCTCTGAAGATGGATCTGG	
*hxk-2*	GCTCTTTAATGGAATTGGCTCG	CGCAATCGTTTCGAGAGTCA	
*hxk-3*	CAAAGCAGTGATGAACGACACA	GACACAATTTGATCGGGAAGTCG	
*cyp-35A1*	GTCCACGCTTGATCTGTTCC	CTCCAGTGACTACCGTTCGT	*
*cyp-35A3*	GCAGATAAACTACACGCCCC	TGTGCTACAGTGACTCCGTT	
*cyp-35B2*	CTGTGAACGCTGAGAATCCG	CGTGAGCCATTTTCCGTGAT	
*cyp-35C1*	TAACCGGCCAAGAAACAACG	TGAGGATGGATGCATGTCGT	

* Primer sequences were designed by our laboratory team; ACTB—β-actin; CCND—cyclin D; AHR—aryl hydrocarbon receptor; CYP—cytochrome P450; *act*—actin; *hxk*—hexokinase; *cyp*—cytochrome P450.

**Table 2:** Proposed phytochemical constituents in the ethanolic extract of *Aquilaria crassna* (ACEE) compared with the National Institute of Standards and Technology (NIST) database.

Compound	RT	Area (%)	MF	MW
Glyceraldehyde	2.871	0.62	C_3_H_6_O_3_	90
3′Hydroxyacetophenone	17.056	0.28	C_8_H_8_O_2_	136
4-Hydroxybenzoic acid	18.612	0.51	C_7_H_6_O_3_	138
(E)-4-(3-Hydroxyprop-1-en-1-yl)-2-methoxyphenol	23.897	0.07	C_10_H_12_O_3_	180
Tetradecanoic acid	24.359	0.07	C_14_H_28_O_2_	228
6-Hydroxy-4,4,7a-trimethyl-5,6,7,7a-tetrahydrobenzofuran-2(4H)-one	24.518	0.19	C_11_H_16_O_3_	196
Neophytadiene	26.03	8.53	C_20_H_38_	278
3,7,11,15-Tetramethylhexadec-2-ene	26.172	0.74	C_20_H_40_	280
3,7,11,15-Tetramethyl-2-hexadecen-1-ol (Phytol)	26.890	2.43	C_20_H_40_O	296
n-Hexadecanoic acid	28.430	1.01	C_16_H_32_O_2_	256
trans-Sinapyl alcohol	29.001	0.11	C_11_H_14_O_4_	210
Hexadecanoic acid, ethyl ester	29.070	0.08	C_18_H_36_O_2_	284
Linoleic acid ethyl ester	32.253	0.05	C_20_H_36_O_2_	308
Hexadecanamide	32.418	0.1	C_16_H_33_NO	255
9-Octadecenamide, (Z)-	35.489	1.58	C_18_H_35_NO	281
Squalene	42.527	23.1	C_30_H_50_	410
Vitamin E	46.533	10.00	C_29_H_50_O_2_	430
Clionasterol	48.825	5.85	C_29_H_50_O	414
β-Amyrone	48.968	5.85	C_30_H_48_O	424
β-Amyrin	49.285	4.05	C_30_H_50_O	426
Lupenone	49.603	6.6	C_30_H_48_O	424
Friedelan-3-one	51.653	12.76	C_30_H_50_O	426
Acetyleburicoic acid	51.979	6.05	C_33_H_52_O_4_	512

RT—retention time; MF—molecular formula; MW—molecular weight.

**Table 3:** The percentage of cell numbers measured by analytical flow cytometry.

	0.1% DMSO (%)	40 µM B[a]P (%)	40 µM B[a]P + 5 µg/mL ACEE (%)
G0/G1	49.84 ± 3.02	40.83 ± 1.05 *	50.49 ± 0.95 ^#^
S	16.32 ± 1.26	12.55 ± 1.62	16.65 ± 1.71
G_2_M	16.76 ± 8.58	17.33 ± 1.89	15.29 ± 1.05

Data are presented as the mean ± SEM, * *p* < 0.05 vs. control and ^#^ *p* < 0.05 vs. 40 µM B[a]P alone.

**Table 4:** Results and statistical analyses of lifespan of *C. elegans* treated with high B[a]P and *Aquilaria crassna* ethanolic extract (ACEE).

Group	Mean Lifespan		*p*-Value (vs. 40 µM B[a]P)	Number of Worms
	Day ± SEM	% Increase (vs. 40 µM B[a]P)		
0.1% DMSO	15.40 ± 0.26	13.23	0.0169	154
40 µM B[a]P	13.60 ± 0.35	-	-	145
40 µM B[a]P +				
5 µg/mL ACEE	15.42 ± 0.26	13.38	0.0071	173

**Table 5:** Docking results of the compounds with cytochrome P450 1A1 (CYP1A1).

No.	Compound	Binding Energy (kcal/mol)	Inhibition Constant	Amino Acid Interaction		
				Hydrogen Bond	Hydrophobic Bond	Electrostatic Bond
	alpha-naphthoflavone (Original ligand)	−10.16	35.57 nM			
	Alizarin (Positive control)	−8.56	535.22 nM	SER116 ASN255	ILE115 PHE224 (3)	
	Purpurin (Positive control)	−8.97	267.12 nM	SER116 ASN255 (2) LEU312	ILE115 PHE224 (3) PHE258 (3) LEU312	
1	Neophytadiene	−7.83	1.83 µM		ILE115 PHE123 PHE224 (5) LEU254 PHE258 (3) LEU312 (2) ALA317 (2) PHE319 (2) LEU496	
2	Vitamin E	−10.0	46.57 nM	SER116 GLY316	ILE115 (2) PHE224 (4) PHE258 (3) LEU312 ALA317 (2) PHE319 VAL382 ILE386 CYS457 LEU496 (2)	
3	β-amyrone	−10.12	38.35 nM		ILE115 (2) PHE123 (3) PHE224 (6) PHE258 (2) LEU312 ALA317 (2) PHE319 ILE386	
4	β-amyrin	−1.17	137.92 mM		ILE115 PHE123 (2) PHE224 (2) ALA317 (3) VAL382 (2) ILE386 (3) CYS457 (3) LEU496	
5	Friedelan-3-one	−7.52	3.10 µM	ASP313	PHE224 PHE258 (2) PHE319	
6	Clionasterol	−10.45	22.02 nM		ILE115 PHE123 PHE224 (3) ALA317 (3) VAL382 (2) ILE386 (2) LEU496 (3)	
7	Squalene	−8.92	287.33 nM		ILE115 PHE123 PHE224 (3) PHE258 (2) LEU312 (2) ALA317 (4) PHE319 ILE386 CYS457 (3) ILE458 LEU496	
8	3,7,11,15-Tetramethyl-2-hexadecen-1-ol (Phytol)	−6.81	10.21 µM	PHE258 GLY316	ILE115 (2) PHE123 (2) PHE224 (5) LEU254 PHE258 (2) LEU312 (2) ALA317 (2) LEU496	
9	n-Hexadecanoic acid (Palmitic acid)	−5.28	134.07 µM	SER122	ILE115 PHE123 (2) PHE224 (3) PHE258 LEU312 ALA317 (2) PHE319	
10	9-Octadecenamide, (Z)-	−6.30	24.26 µM	ASP320	ILE115 PHE224 (6) PHE258 ALA317 (2) LEU254 LEU312 PHE319	
11	Lupenone	−6.41	20.16 µM		ILE115 PHE123 PHE224 (2) ALA317 (5) PHE319 LEU496	

**Table 6:** Docking results of the compounds with hexokinase II.

No.	Compound	Binding Energy (kcal/mol)	Inhibition Constant	Amino Acid Interaction		
				Hydrogen Bond	Hydrophobic Bond	Electrostatic Bond
	2-deoxy-2-{[(2E)-3-(3,4-dichlorophenyl)prop-2-enoyl]amino}7-alpha-D-glucopyranose (Original ligand)	−6.52	16.65 µM			
	Metrizamide (Positive control)	−6.53	16.40 µM	LYS62 CYS158 GLU260 ASN287 GLU294 (2)	PRO157 (2)	GLU260
	Lonidamine (Positive control)	−4.58	441.25 µM	CYS158 GLU260	PHE154 PRO157 (4) CYS158 (2)	
1	Neophytadiene	−4.48	515.98 µM		PHE154 PRO157 CYS158 (2) VAL206 ILE229	
2	Vitamin E	−6.43	19.36 µM	CYS158	PRO157 (4) CYS158 ILE229 (2) MET300	GLU260
3	β-amyrone	−6.51	16.95 µM		PRO157 (2) CYS158 VAL206	
4	β-amyrin	−7.46	3.42 µM	ASP209 ASN235	PRO157 ILE229	
5	Friedelan-3-one	−4.96	231.14 µM	ASN208 ASN235		
6	Clionasterol	−6.64	13.56 µM		LYS62 LEU64 (3) PRO157 (3) CYS158 ALA263 LYS290 LYS290	
7	Squalene	−4.20	840.58 µM		LYS62 (2) LEU64 (2) PHE154 PRO157 (4) CYS158 (2) VAL206 ALA263 (2)	
8	3,7,11,15-Tetramethyl-2-hexadecen-1-ol (Phytol)	−3.58	2.38 mM	GLU260	LYS62 LEU64 PRO157 (4) ALA263	
9	n-Hexadecanoic acid (Palmitic acid)	−2.31	20.31 mM	THR232	ILE229	
10	9-Octadecenamide, (Z)-	−3.86	1.49 mM	ASN208 ASN235 GLU260	LYS62 (2) LEU64 LEU64 PRO157 (3) CYS158 ALA263	
11	Lupenone	−7.05	6.85 µM	ASN208 THR210 ASN235	ILE229 MET300 (2) TYR301	

**Table 7:** Bioactivity of phytochemical constituents in *Aquilaria crassna* ethanolic extract (ACEE).

Compound	Bioactivity
4-Hydroxybenzoic acid	Anti-inflammatory activities [50] Antioxidant activities [51,52,53]
Tetradecanoic acid	Some monohydroxy tetradecanoic acid isomers provide urease and elastase inhibitors, and antioxidants [54]
6-Hydroxy-4,4,7a-trimethyl-5,6,7,7a-tetrahydrobenzofuran-2(4H)-one	Anti-inflammatory activity [55]
Neophytadiene	Antimicrobial, antifungal, anti-inflammatory and antioxidant activities [56]
3,7,11,15-Tetramethyl-2-hexadecen-1-ol (Phytol)	Antioxidant and neuroprotective effects [57,58] Anticholinesterase activity [59]
n-Hexadecanoic acid (Palmitic acid)	Anti-inflammatory activities Antioxidant activities [60,61]
trans-Sinapyl alcohol	Anti-inflammatory and antinociceptive activities [62]
Hexadecanoic acid, ethyl ester (Ethyl palmitate)	Anti-inflammatory activities [63]
Linoleic acid ethyl ester	Anti-inflammatory activities [64]
9-Octadecenamide, (Z)-(Oleamide)	Anti-inflammatory activities [65] Chemoprotective agent against Alzheimer’ s disease [66,67]
Squalene	Antioxidant activity [68]
Vitamin E	Antioxidant activity [63,69]
Clionasterol	Anticomplementary effect [70] Inhibition of particulate matter-induced oxidative stress and apoptosis in skin [71]
β-Amyrone	Anti-inflammatory activity [72]
β-Amyrin	Anti-inflammatory activity [73]
Lupenone	Anti-inflammatory activity [74,75] Anticancer [76,77]
Friedelan-3-one	Antimicrobial properties [78]
Acetyleburicoic acid	Antidiabetic and antihyperlipidemic effects [79]

## Data Availability

Not applicable.

## References

[1] De Rubis G., Paudel K.R., Manandhar B., Singh S.K., Gupta G., Malik R., Shen J., Chami A., MacLoughlin R., Chellappan D.K. (2023). Agarwood Oil Nanoemulsion Attenuates Cigarette Smoke-Induced Inflammation and Oxidative Stress Markers in BCi-NS1. 1 Airway Epithelial Cells. Nutrients.

[2] Wongwad E., Pingyod C., Saesong T., Waranuch N., Wisuitiprot W., Sritularak B., Temkitthawon P., Ingkaninan K. (2019). Assessment of the bioactive components, antioxidant, antiglycation and anti-inflammatory properties of *Aquilaria crassna* Pierre ex Lecomte leaves. Ind. Crops Prod..

[3] Pattarachotanant N., Sornkaew N., Warayanon W., Rangsinth P., Sillapachaiyaporn C., Vongthip W., Chuchawankul S., Prasansuklab A., Tencomnao T. (2022). Aquilaria crassna leaf extract ameliorates glucose-induced neurotoxicity in vitro and improves lifespan in caenorhabditis elegans. Nutrients.

[4] Lamptey R.N., Chaulagain B., Trivedi R., Gothwal A., Layek B., Singh J. (2022). A review of the common neurodegenerative disorders: Current therapeutic approaches and the potential role of nanotherapeutics. Int. J. Mol. Sci..

[5] Merelli A., Czornyj L., Lazarowski A. (2013). Erythropoietin: A neuroprotective agent in cerebral hypoxia, neurodegeneration, and epilepsy. Curr. Pharm. Des..

[6] Boström C.-E., Gerde P., Hanberg A., Jernström B., Johansson C., Kyrklund T., Rannug A., Törnqvist M., Victorin K., Westerholm R. (2002). Cancer risk assessment, indicators, and guidelines for polycyclic aromatic hydrocarbons in the ambient air. Environ. Health Perspect..

[7] Bukowska B., Mokra K., Michałowicz J. (2022). Benzo [a] pyrene—Environmental occurrence, human exposure, and mechanisms of toxicity. Int. J. Mol. Sci..

[8] Hardonnière K., Saunier E., Lemarié A., Fernier M., Gallais I., Héliès-Toussaint C., Mograbi B., Antonio S., Bénit P., Rustin P. (2016). The environmental carcinogen benzo [a] pyrene induces a Warburg-like metabolic reprogramming dependent on NHE1 and associated with cell survival. Sci. Rep..

[9] Tancell P., Rhead M., Trier C., Bell M., Fussey D. (1995). The sources of benzo [a] pyrene in diesel exhaust emissions. Sci. Total Environ.

[10] Bukowska B., Sicińska P. (2021). Influence of benzo (a) pyrene on different epigenetic processes. Int. J. Mol. Sci..

[11] Knafla A., Phillipps K., Brecher R., Petrovic S., Richardson M. (2006). Development of a dermal cancer slope factor for benzo [a] pyrene. Regul. Toxicol. Pharmacol..

[12] Das S.K., Patel B., Patri M. (2016). Neurotoxic effect of benzo [a] pyrene and its possible association with 6-hydroxydopamine induced neurobehavioral changes during early adolescence period in rats. J. Toxicol..

[13] Jorge B.C., Reis A.C.C., Stein J., da Silva Balin P., Sterde E.T., Barbosa M.G., de Aquino A.M., Kassuya C.A.L., Arena A.C. (2021). Parental exposure to benzo (a) pyrene in the peripubertal period impacts reproductive aspects of the F1 generation in rats. Reprod. Toxicol..

[14] Saunders C.R., Das S.K., Ramesh A., Shockley D.C., Mukherjee S. (2006). Benzo (a) pyrene–induced acute neurotoxicity in the F–344 rat: Role of oxidative stress. J. Appl. Toxicol. Int. J..

[15] Menzel R., Bogaert T., Achazi R. (2001). A systematic gene expression screen of Caenorhabditis elegans cytochrome P450 genes reveals CYP35 as strongly xenobiotic inducible. Arch. Biochem. Biophys..

[16] Menzel R., Rödel M., Kulas J., Steinberg C.E. (2005). CYP35: Xenobiotically induced gene expression in the nematode *Caenorhabditis elegans*. Arch. Biochem. Biophys..

[17] Pattarachotanant N., Tencomnao T. (2020). Citrus hystrix extracts protect human neuronal cells against high glucose-induced senescence. Pharmaceuticals.

[18] Prasansuklab A., Meemon K., Sobhon P., Tencomnao T. (2017). Ethanolic extract of Streblus asper leaves protects against glutamate-induced toxicity in HT22 hippocampal neuronal cells and extends lifespan of *Caenorhabditis elegans*. BMC Complement. Altern. Med..

[19] Singleton V.L., Orthofer R., Lamuela-Raventós R.M. (1999). [14] Analysis of total phenols and other oxidation substrates and antioxidants by means of folin-ciocalteu reagent. Methods in Enzymology.

[20] Hasler A., Sticher O., Meier B. (1992). Identification and determination of the flavonoids from Ginkgo biloba by high-performance liquid chromatography. J. Chromatogr. A.

[21] Zhishen J., Mengcheng T., Jianming W. (1999). The determination of flavonoid contents in mulberry and their scavenging effects on superoxide radicals. Food Chem..

[22] Williams D.E. (1966). Structure of 2,2-Diphenyl-1-picrylhydrazyl Free Radical1. J. Am. Chem. Soc..

[23] Bourbonnais R., Leech D., Paice M.G. (1998). Electrochemical analysis of the interactions of laccase mediators with lignin model compounds. BBA—Gen. Subj..

[24] Septisetyani E.P., Ningrum R.A., Romadhani Y., Wisnuwardhani P.H., Santoso A. (2014). Optimization of sodium dodecyl sulphate as a formazan solvent and comparison of 3-(4,-5-dimethylthiazo-2-yl)-2, 5-diphenyltetrazolium bromide (MTT) assay with wst-1 assay in mcf-7 cells. Indones. J. Pharm..

[25] Pattarachotanant N., Rakkhitawatthana V., Tencomnao T. (2014). Effect of *Gloriosa superba* and *Catharanthus roseus* extracts on IFN-γ-induced keratin 17 expression in HaCaT human keratinocytes. eCAM.

[26] Rangsinth P., Prasansuklab A., Duangjan C., Gu X., Meemon K., Wink M., Tencomnao T. (2019). Leaf extract of Caesalpinia mimosoides enhances oxidative stress resistance and prolongs lifespan in *Caenorhabditis elegans*. BMC Complement. Altern. Med..

[27] Kaewjanthong P., Sooksai S., Sasano H., Hutvagner G., Bajan S., McGowan E., Boonyaratanakornkit V. (2022). Cell-penetrating peptides containing the progesterone receptor polyproline domain inhibits EGF signaling and cell proliferation in lung cancer cells. PLoS ONE.

[28] Malar D.S., Prasanth M.I., Verma K., Prasansuklab A., Tencomnao T. (2022). Hibiscus sabdariffa Extract Protects HaCaT Cells against Phenanthrene-Induced Toxicity through the Regulation of Constitutive Androstane Receptor/Pregnane X Receptor Pathway. Nutrients.

[29] Gu Q.L., Zhang Y., Fu X.M., Lu Z.L., Yu Y., Chen G., Ma R., Kou W., Lan Y.M. (2020). Toxicity and metabolism of 3-bromopyruvate in *Caenorhabditis elegans*. J. Zhejiang Univ. Sci. B.

[30] Walsh A.A., Szklarz G.D., Scott E.E. (2013). Human cytochrome P450 1A1 structure and utility in understanding drug and xenobiotic metabolism. J. Biol. Chem..

[31] Lin H., Zeng J., Xie R., Schulz M.J., Tedesco R., Qu J., Erhard K.F., Mack J.F., Raha K., Rendina A.R. (2016). Discovery of a Novel 2,6-Disubstituted Glucosamine Series of Potent and Selective Hexokinase 2 Inhibitors. ACS Med. Chem. Lett..

[32] Pattarachotanant N., Prasansuklab A., Tencomnao T. (2021). Momordica charantia L. Extract Protects Hippocampal Neuronal Cells against PAHs-Induced Neurotoxicity: Possible Active Constituents Include Stigmasterol and Vitamin E. Nutrients.

[33] Chaikhong K., Chumpolphant S., Rangsinth P., Sillapachaiyaporn C., Chuchawankul S., Tencomnao T., Prasansuklab A. (2023). Antioxidant and Anti-Skin Aging Potential of Selected Thai Plants: In Vitro Evaluation and In Silico Target Prediction. Plants.

[34] Takahashi E., Fujita K.-I., Kamataki T., Arimoto-Kobayashi S., Okamoto K., Negishi T. (2002). Inhibition of human cytochrome P450 1B1, 1A1 and 1A2 by antigenotoxic compounds, purpurin and alizarin. Mutat. Res. Fundam. Mol..

[35] Bertoni J.M. (1982). Metrizamide inhibits human brain hexokinase. Neurology.

[36] Huang Y., Sun G., Sun X., Li F., Zhao L., Zhong R., Peng Y. (2020). The potential of lonidamine in combination with chemotherapy and physical therapy in cancer treatment. Cancers.

[37] Nath K., Guo L., Nancolas B., Nelson D.S., Shestov A.A., Lee S.-C., Roman J., Zhou R., Leeper D.B., Halestrap A.P. (2016). Mechanism of antineoplastic activity of lonidamine. BBA-Rev. Cancer.

[38] Begriche K., Massart J., Fromenty B. (2019). Mitochondrial dysfunction induced by xenobiotics: Involvement in steatosis and steatohepatitis. Mitochondria in Obesity and Type 2 Diabetes.

[39] Gao D., Wu M., Wang C., Wang Y., Zuo Z. (2015). Chronic exposure to low benzo [a] pyrene level causes neurodegenerative disease-like syndromes in zebrafish (*Danio rerio*). Aquat. Toxicol..

[40] Manzella C., Singhal M., Alrefai W.A., Saksena S., Dudeja P.K., Gill R.K. (2018). Serotonin is an endogenous regulator of intestinal CYP1A1 via AhR. Sci. Rep..

[41] Kabadi S.V., Stoica B.A., Loane D.J., Byrnes K.R., Hanscom M., Cabatbat R.M., Tan M.T., Faden A.I. (2012). Cyclin D1 gene ablation confers neuroprotection in traumatic brain injury. J. Neurotrauma.

[42] Abbass M., Chen Y., Arlt V.M., Stürzenbaum S.R. (2021). Benzo [a] pyrene and Caenorhabditis elegans: Defining the genotoxic potential in an organism lacking the classical CYP1A1 pathway. Arch. Toxicol..

[43] Aarnio V., Lehtonen M., Storvik M., Callaway J.C., Lakso M., Wong G. (2011). Caenorhabditis elegans mutants predict regulation of fatty acids and endocannabinoids by the CYP-35A gene family. Front. Pharmacol..

[44] Imanikia S., Hylands P., Stürzenbaum S.R. (2015). The double mutation of cytochrome P450’s and fatty acid desaturases affect lipid regulation and longevity in *C. elegans*. Biochem. Biophys..

[45] Zhang Y., Zou X., Ding Y., Wang H., Wu X., Liang B. (2013). Comparative genomics and functional study of lipid metabolic genes in Caenorhabditis elegans. BMC Genom..

[46] Thies J.L., Willicott K., Craig M.L., Greene M.R., DuGay C.N., Caldwell G.A., Caldwell K.A. (2023). Xanthine Dehydrogenase Is a Modulator of Dopaminergic Neurodegeneration in Response to Bacterial Metabolite Exposure in *C. elegans*. Cells.

[47] Liu R., Liu X. (2022). Virtual Screening and Biological Activity Evaluation of New Potent Inhibitors Targeting Hexokinase-II. Molecules.

[48] Wilson J.E. (2003). Isozymes of mammalian hexokinase: Structure, subcellular localization and metabolic function. J. Exp. Biol..

[49] de Meis L., Grieco M.A.B., Galina A. (1992). Reversal of oxidative phosphorylation in submitochondrial particles using glucose 6-phosphate and hexokinase as an ATP regenerating system. FEBS Lett..

[50] Luecha P., Umehara K., Miyase T., Noguchi H. (2009). Antiestrogenic constituents of the Thai medicinal plants Capparis flavicans and Vitex glabrata. J. Nat. Prod..

[51] Herrmann K., Nagel C.W. (1989). Occurrence and content of hydroxycinnamic and hydroxybenzoic acid compounds in foods. Crit. Rev. Food Sci..

[52] Pulido R., Bravo L., Saura-Calixto F. (2000). Antioxidant activity of dietary polyphenols as determined by a modified ferric reducing/antioxidant power assay. J. Agric. Food Chem..

[53] Rice-Evans C.A., Miller N.J., Paganga G. (1996). Structure-antioxidant activity relationships of flavonoids and phenolic acids. Free Radic. Biol. Med..

[54] Sokmen B.B., Hasdemir B., Yusufoglu A., Yanardag R. (2014). Some monohydroxy tetradecanoic acid isomers as novel urease and elastase inhibitors and as new antioxidants. Appl. Biochem. Biotechnol..

[55] Jayawardena T.U., Kim H.-S., Sanjeewa K.A., Kim S.-Y., Rho J.-R., Jee Y., Ahn G., Jeon Y.-J. (2019). Sargassum horneri and isolated 6-hydroxy-4, 4, 7a-trimethyl-5, 6, 7, 7a-tetrahydrobenzofuran-2 (4H)-one (HTT); LPS-induced inflammation attenuation via suppressing NF-κB, MAPK and oxidative stress through Nrf2/HO-1 pathways in RAW 264.7 macrophages. Algal Res..

[56] Raman B.V., Samuel L., Saradhi M.P., Rao B.N., Krishna N., Sudhakar M., Radhakrishnan T. (2012). Antibacterial, antioxidant activity and GC-MS analysis of Eupatorium odoratum. Asian J. Pharm. Clin. Res..

[57] Silva R.O., Sousa F.B.M., Damasceno S.R., Carvalho N.S., Silva V.G., Oliveira F.R.M., Sousa D.P., Aragão K.S., Barbosa A.L., Freitas R.M. (2014). Phytol, a diterpene alcohol, inhibits the inflammatory response by reducing cytokine production and oxidative stress. Fundam. Clin. Pharmacol..

[58] Syad A.N., Rajamohamed B.S., Shunmugaiah K.P., Kasi P.D. (2016). Neuroprotective effect of the marine macroalga Gelidiella acerosa: Identification of active compounds through bioactivity-guided fractionation. Pharm. Biol..

[59] Elufioye T.O., Obuotor E.M., Agbedahunsi J.M., Adesanya S.A. (2017). Anticholinesterase constituents from the leaves of *Spondias mombin* L.(Anacardiaceae). Biol. Targets Ther..

[60] Çakmak Y.S., Aktumsek A., Duran A. (2012). Studies on antioxidant activity, volatile compound and fatty acid composition of different parts of *Glycyrrhiza echinata* L. EXCLI J..

[61] Tyagi T., Agarwal M. (2017). Phytochemical screening and GC-MS analysis of bioactive constituents in the ethanolic extract of *Pistia stratiotes* L. and *Eichhornia crassipes* (Mart.) solms. J. Pharmacogn. Phytochem..

[62] Choi J., Shin K.-M., Park H.-J., Jung H.-J., Kim H.J., Lee Y.S., Rew J.-H., Lee K.-T. (2004). Anti-inflammatory and antinociceptive effects of sinapyl alcohol and its glucoside syringin. Planta Med..

[63] Saeed N.M., El-Demerdash E., Abdel-Rahman H.M., Algandaby M.M., Al-Abbasi F.A., Abdel-Naim A.B. (2012). Anti-inflammatory activity of methyl palmitate and ethyl palmitate in different experimental rat models. Toxicol. Appl. Pharmacol..

[64] Kolar M.J., Konduri S., Chang T., Wang H., McNerlin C., Ohlsson L., Härröd M., Siegel D., Saghatelian A. (2019). Linoleic acid esters of hydroxy linoleic acids are anti-inflammatory lipids found in plants and mammals. J. Biol. Chem..

[65] Oh Y.T., Lee J.Y., Lee J., Lee J.H., Kim J.-E., Ha J., Kang I. (2010). Oleamide suppresses lipopolysaccharide-induced expression of iNOS and COX-2 through inhibition of NF-κB activation in BV2 murine microglial cells. Neurosci. Lett..

[66] Ano Y., Ozawa M., Kutsukake T., Sugiyama S., Uchida K., Yoshida A., Nakayama H. (2015). Preventive effects of a fermented dairy product against Alzheimer’s disease and identification of a novel oleamide with enhanced microglial phagocytosis and anti-inflammatory activity. PLoS ONE.

[67] Heo H.-J., Park Y.-J., Suh Y.-M., Choi S.-J., Kim M.-J., Cho H.-Y., Chang Y.-J., Hong B., Kim H.-K., Kim E. (2003). Effects of oleamide on choline acetyltransferase and cognitive activities. Biosci. Biotechnol. Biochem..

[68] Saint–Leger D., Bague A., Lefebvre E., Cohen E., Chivot M. (1986). A possible role for squalene in the pathogenesis of acne. II. In vivo study of squalene oxides in skin surface and intra–comedonal lipids of acne patients. Br. J. Dermatol..

[69] Brigelius-Flohé R. (2006). Bioactivity of vitamin E. Nutr. Res. Rev..

[70] Cerqueira F., Watanadilok R., Sonchaeng P., Kijjoa A., Pinto M., van Ufford H.Q., Kroes B., Beukelman C., Nascimento M.S.J. (2003). Clionasterol: A potent inhibitor of complement component C1. Planta Med..

[71] Liyanage N., Nagahawatta D., Jayawardena T.U., Jayawardhana H., Lee H.-G., Kim Y.-S., Jeon Y.-J. (2022). Clionasterol-Rich Fraction of Caulerpa racemosa against Particulate Matter-Induced Skin Damage via Inhibition of Oxidative Stress and Apoptosis-Related Signaling Pathway. Antioxidants.

[72] Niu X., Yao H., Li W., Mu Q., Li H., Hu H., Li Y., Huang H. (2014). δ-Amyrone, a specific inhibitor of cyclooxygenase-2, exhibits anti-inflammatory effects in vitro and in vivo of mice. Int. Immunopharmacol..

[73] da Silva K.A.S., Paszcuk A.F., Passos G.F., Silva E.S., Bento A.F., Meotti F.C., Calixto J.B. (2011). Activation of cannabinoid receptors by the pentacyclic triterpene α, β-amyrin inhibits inflammatory and neuropathic persistent pain in mice. PAIN.

[74] Romero-Estrada A., Maldonado-Magaña A., González-Christen J., Bahena S.M., Garduño-Ramírez M.L., Rodríguez-López V., Alvarez L. (2016). Anti-inflammatory and antioxidative effects of six pentacyclic triterpenes isolated from the Mexican copal resin of Bursera copallifera. BMC Complement. Altern. Med..

[75] Yasukawa K., Yu S., Yamanouchi S., Takido M., Akihisa T., Tamura T. (1995). Some lupane-type triterpenes inhibit tumor promotion by 12-O-tetradecanoylphorbol-13-acetate in two-stage carcinogenesis in mouse skin. Phytomedicine.

[76] Ahmad S., Sukari M.A., Ismail N., Ismail I.S., Abdul A.B., Abu Bakar M.F., Kifli N., Ee G.C. (2015). Phytochemicals from Mangifera pajang Kosterm and their biological activities. BMC Complement. Altern. Med..

[77] Suwito H., Heffen W.L., Cahyana H., Suwarso W.P. (2016). Isolation, transformation, anticancer, and apoptosis activity of lupeyl acetate from *Artocarpus integra*. AIP Conf. Proc..

[78] Odeh I.C., Tor-Anyiin T.A., Igoli J.O., Anyam J.V. (2016). In vitro antimicrobial properties of friedelan-3-one from Pterocarpus santalinoides L’Herit, ex Dc. Afr. J. Biotechnol..

[79] Lin C.-H., Kuo Y.-H., Shih C.-C. (2017). Eburicoic acid, a triterpenoid compound from Antrodia camphorata, displays antidiabetic and antihyperlipidemic effects in palmitate-treated C2C12 myotubes and in high-fat diet-fed mice. Int. J. Mol. Sci..

[80] Zhang Z., Yi P., Yang J., Huang J., Xu P., Hu M., Zhang C., Wang B., Peng W. (2020). Integrated network pharmacology analysis and serum metabolomics to reveal the cognitive improvement effect of Bushen Tiansui formula on Alzheimer’s disease. J. Ethnopharmacol..

